# Vascular Dysfunction Induced in Offspring by Maternal Dietary Fat Involves Altered Arterial Polyunsaturated Fatty Acid Biosynthesis

**DOI:** 10.1371/journal.pone.0034492

**Published:** 2012-04-03

**Authors:** Christopher J. Kelsall, Samuel P. Hoile, Nicola A. Irvine, Mojgan Masoodi, Christopher Torrens, Karen A. Lillycrop, Philip C. Calder, Geraldine F. Clough, Mark A. Hanson, Graham C. Burdge

**Affiliations:** 1 Academic Unit of Human Development and Health, Faculty of Medicine, University of Southampton, Hampshire, United Kingdom; 2 MRC Human Nutrition Research, Elsie Widdowson Laboratory, Cambridge, United Kingdom; 3 Faculty of Natural and Environmental Sciences, University of Southampton, Hampshire, United Kingdom; Fundação Oswaldo Cruz, Brazil

## Abstract

Nutrition during development affects risk of future cardiovascular disease. Relatively little is known about whether the amount and type of fat in the maternal diet affect vascular function in the offspring. To investigate this, pregnant and lactating rats were fed either 7%(w/w) or 21%(w/w) fat enriched in either18:2n-6, *trans* fatty acids, saturated fatty acids, or fish oil. Their offspring were fed 4%(w/w) soybean oil from weaning until day 77. Type and amount of maternal dietary fat altered acetylcholine (ACh)-mediated vaso-relaxation in offspring aortae and mesenteric arteries, contingent on sex. Amount, but not type, of maternal dietary fat altered phenylephrine (Pe)-induced vasoconstriction in these arteries. Maternal 21% fat diet decreased 20:4n-6 concentration in offspring aortae. We investigated the role of Δ6 and Δ5 desaturases, showing that their inhibition in aortae and mesenteric arteries reduced vasoconstriction, but not vaso-relaxation, and the synthesis of specific pro-constriction eicosanoids. Removal of the aortic endothelium did not alter the effect of inhibition of Δ6 and Δ5 desaturases on Pe-mediated vasoconstriction. Thus arterial smooth muscle 20:4n-6 biosynthesis *de novo* appears to be important for Pe-mediated vasoconstriction. Next we studied genes encoding these desaturases, finding that maternal 21% fat reduced *Fads2* mRNA expression and increased *Fads1* in offspring aortae, indicating dysregulation of 20:4n-6 biosynthesis. Methylation at CpG −394 bp 5′ to the *Fads2* transcription start site predicted its expression. This locus was hypermethylated in offspring of dams fed 21% fat. Pe treatment of aortae for 10 minutes increased *Fads2*, but not *Fads1*, mRNA expression (76%; P<0.05). This suggests that *Fads2* may be an immediate early gene in the response of aortae to Pe. Thus both amount and type of maternal dietary fat induce altered regulation of vascular tone in offspring though differential effects on vaso-relaxation, and persistent changes in vasoconstriction via epigenetic processes controlling arterial polyunsaturated fatty acid biosynthesis.

## Introduction

Endothelial dysfunction is a critical mechanism in the pathogenesis of hypertension and in atherogenesis [1], [2]. Endothelial dysfunction results in a reduced response to vasodilators, including nitric oxide (NO) and specific eicosanoids, and enhanced responses to endothelium-derived constricting factors including endothelin-1, prostaglandin (PG) E_2_ and F_2α_ and thromboxane (TBX) A_2_ that counteract the effects of endothelium-derived vasodilators [3]. There is direct evidence from spontaneously hypertensive rats and indirect evidence from human studies that enhanced synthesis of arachidonic acid (20:4n-6) metabolites is a major causal factor in endothelial dysfunction [3]. Hence capacity to supply 20:4n-6 for synthesis of vasoactive eicosanoids is of potential importance in the regulation of vascular tone.

Dietary fatty acid intake has been shown to induce changes in endothelial function and risk of cardiovascular disease (CVD). High total fat intake, particularly of saturated fatty acids (SFA) or *trans* fatty acids (TFA) increases risk of hypertension and CVD [4], whilst dietary supplementation with fish oils containing the n-3 PUFA eicosapentaenoic acid (20:5n-3) and docosahexaenoic acid (22:6n-3) has beneficial effects [5]; it is less clear whether similar effects are associated with higher 18:3n-3 status or dietary supplementation [6]. Higher intakes of n-6 polyunsaturated fatty acids (PUFA), predominately linoleic acid (18:2n-6), have variable effects on cardiovascular health [7]. 20:4n-6 is synthesised from 18:2n-6 by sequential actions of Δ6 and Δ5 desaturases [8]. Thus one possible mechanism by which differences in 18:2n-6 intake may alter CVD risk is altered synthesis of 20:4n-6 and it has been proposed that impaired Δ6 and Δ5 desaturase activity may contribute to atherosclerosis [9]. This is supported by the associations between polymorphisms in *Fads1* and *Fads2* which encode Δ5 and Δ6 desaturases, respectively, and risk of CVD [10]. This implies that both dietary fat intake and capacity for synthesis of specific PUFA *de novo* are important for cardiovascular health. The precise mechanism that links *Fads1* and *Fads2* polymorphisms to impaired vascular function is not understood completely and may involve altered synthesis of vasoactive agents and inflammatory mediators [11], and modulation of blood lipid concentrations [11], [12]. Because such polymorphisms alter the fatty acid composition of blood lipids it is generally assumed that affects on cardiovascular health are due primarily to changes in the supply of PUFA from the liver to peripheral vessels. However, PUFA biosynthesis has been demonstrated in isolated arterial endothelial [13] and smooth muscle [14] cells, but the contribution of this pathway to vascular function is not known.

Unbalanced nutrition in early life increases future risk of non-communicable diseases including CVD [15]. Although pregnant and nursing women exhibit the same trends for increasing fat intake and dietary choices as the rest of the population, relatively little is known about the effects of differences in maternal dietary fat on cardiovascular function on their children. However, studies in animal models support the view that maternal fat intake can induce persistent changes in vascular function in the offspring. For example, feeding rats a high SFA diet during pregnancy and lactation induced hypertension in the offspring [16], impaired endothelium-derived hyperpolarising factor activity [17], and reduced endothelial cell volume and smooth muscle cell number [18], and aortic Na+/K+ ATPase activity [19]. Dietary n-3 PUFA deficiency during the perinatal period in rats also induced hypertension in the offspring which was exacerbated by continued n-3 PUFA deficiency after weaning, but was partially ameliorated by supplementation with fish oil [20].

The mechanism by which high maternal intake of saturated fat or n-3 PUFA deficiency induce in the offspring persistent changes in pathways which regulate vascular tone has not been described. Previous studies have shown that protein restriction [21], global under nutrition [22], or supplementation with methyl donors [23] in dams, or neonatal over nutrition [24] induce altered epigenetic regulation of specific genes in adult offspring. Therefore, altered epigenetic regulation of specific genes represents one potential mechanism by which maternal fat intake induces long-term changes in vascular function in the offspring.

We therefore investigated the effect of different types and amounts, and of any interactions between these factors, of maternal dietary fat on vascular function in the adult offspring. We assessed vascular function in the thoracic aorta, a conduit artery, and in mesenteric arteries, resistance vessels, by measuring the response to the muscarinic receptor agonist acetylcholine (ACh) and the α_1_-adrenergic receptor agonist phenylephrine (Pe).

## Materials and Methods

### Ethical statement

All animal procedures were in accordance with the British Home Office Animals (Scientific Procedures) Act (1986) and were conducted under Home Office Licence number 70–6457. The study received institutional approval from the University of Southampton Biomedical Research Facility Research Ethics Committee. Ethical approval for a proof-of-concept study using discarded arteries from amputations was granted by the Clinical Governance Office, University Hospital Southampton NHS Foundation Trust. Patients provided informed, written consent.

### Animal Procedures

Virgin female Wistar rats weighing 200–250 g were fed on diets based on the AIN93G formulation [25] and containing either 7% or 21% (w/w) total fat (Table S1) from 14 days before conception, and throughout pregnancy and lactation. The fat component of these diets was either safflower oil SAO (enriched in 18:2n-6), hydrogenated soybean oil (HSO) enriched in TFA, butter enriched in saturated fatty acids, or Menhaden oil (MO) enriched in 20:5n-3 and 22:6n-3. All diets contained the same amount of vitamin E to provide sufficient anti-oxidant capacity at the highest level of MO intake. Female rats were mated with randomly assigned males, day 0 of pregnancy being defined by plug detection. Litters were culled within 24 h of spontaneous birth to 8 pups (equal numbers of males and females). Offspring were weaned at 28 days onto on AIN-93M (Table S1) and maintained on this diet until day 77 when they were fasted for 12 hours and killed by CO_2_ asphyxiation and cervical dislocation.

### Measurement of Vascular Function Ex-Vivo

The effect of differences in type and amount of maternal dietary fat on markers of endothelial function, ACh-induced vaso-relaxation and Pe-induced vaso-constriction, were investigated in aortae and mesenteric arteries in adult offspring on day 77 by wire myography [26]. Rat thoracic aortae and small mesenteric arteries (internal diameter ca. 250 µm) were dissected and placed in cold (4°C) physiological salt solution (PSS; NaCl, 119; KCl, 4.7; CaCl_2_, 2.5; MgSO_4_, 1.17; NaHCO_3_, 25; KH_2_PO_4_, 1.18; EDTA, 0.026; and D-glucose, 5.5 mM). Segments from rat aortae and mesenteric arteries, and human femoral arteries were cleaned of connective tissue, mounted in a wire myograph (Danish Myo Technology A/S, Denmark) and bathed in PSS at 37°C and continuously gassed with 95% O_2_ and 5% CO_2_ [26].

Segments were normalised and functional integrity of mesenteric segments was assessed by 2 minute incubations with 125 mM K^+^ in physiological saline solution (KPSS) [26]. Mesenteric segments failing to produce an active tension equivalent to 13.3 kPa were discarded. Following normalisation, cumulative concentration response curves were constructed to the α_1_-adrenoceptor agonist Pe (10 nM–100 µM). Vessels were pre-constricted with a sub-maximal dose of Pe equivalent to pEC_80_ (effective concentration equal to 80% of maximal concentration) and responses were measured to the endothelium-dependent vasodilator ACh (0.1 nM–10 µM). Constrictor responses were calculated as % maximum contraction induced by 125 mM KPSS and relaxant responses as % reversal of Pe-induced contraction [26].

In order to determine whether PUFA biosynthesis *de novo* may be involved in the regulation of vascular tone, aorta and mesenteric arteries from adult male rats which had not been exposed to altered nutrition during development were treated with the specific Δ6 and Δ5 desaturase inhibitors 2,2-diphenyl-5-(4-[[(1 E)-pyridin-3-yl-methylidene]amino]piperazin-1-yl)pentanenitrile (SC-26196) [27] or sesamin [28], respectively. All vessel segments were incubated with these inhibitors (each at 10 nM, 1 µM and 100 µM) for 30 minutes prior to assessment of Pe-mediated constriction or ACh-induced vaso-relaxation.

In order to determine whether PUFA biosynthesis was active in arterial endothelial or smooth muscle cells, the constriction response to Pe with or without SC26196 (10^−4^ M) or sesamin (10^−4^ M) was determined in whole aortae or after removal of the endothelium by a modification described by [29]. Aortae were dissected and the response to Pe with or without SC26196 or sesamin determined as above. The endothelium was removed from sections of the same vessel by sliding a cotton thread through the vessel. Removal of the endothelium was assessed by an increase in response to Pe [29] and abolition of response to ACh (0.1 nM–10 µM). The vasoconstriction response to Pe with or without SC26196 or sesamin was then determined.

Cumulative concentration response curves to agonists were analysed by fitting to a 4-parameter logistic equation using nonlinear regression to obtain the effective concentration equal to 50% of maximum (pEC_50_) (Prism 5.0, GraphPAD Software Inc.) [26]. Values from individual samples were used for statistical analysis.

### Analysis of Vasoconstriction in Human Femoral Arteries

Femoral arteries were collected from two subjects undergoing leg amputation for atherosclerotic disease. Regions of apparently uninvolved artery were dissected, placed in cold PSS (4°C) and processed for myography as described below.

### Analysis of Rat Aorta and Plasma Fatty Acid Compositions by Gas Chromatography

Plasma and aorta fatty acid compositions were measured as described [30]. Aortae (approximately 100 mg) were powdered under liquid nitrogen and total lipids extracted with chloroform and methanol [31]. Plasma (0.8 ml) was extracted with chloroform and methanol [31] and then fractionated into individual lipid classes by solid phase extraction using 100 mg aminopropyl silica cartridges [30]. Lipid fractions from aorta and plasma were converted to fatty acid methyl esters (FAMEs) by incubation with methanol containing 2% (v/v) sulphuric acid (56). FAMEs were recovered by extraction with hexane [30]. The proportions of individual fatty acids were measured by gas chromatography using BPX70 fused silica capillary column (30 m×0.25 mm×0.25 µm) on an Aglient 6890 gas chromatograph equipped with flame ionisation detection [30]. FAMEs were identified by their retention times relative to standards and quantified using Chemstation software (Agilent).

### Quantitative Real-Time RTPCR

Measurement of the levels of specific mRNA transcripts was carried out as described [32]. Total RNA was isolated from aorta using Tri Reagent (Sigma) according to the manufacturer's instructions. cDNA was prepared [21] and amplified using real-time RT-PCR [33]. Primer sets were: *Fads1* QuantiTect assay QT00188664 (Qiagen), *Fads2* QuantiTect assay QT00186739 and *eNOS* QuantiTect assay QT01570618. Samples were analysed in duplicate and expression of the individual transcripts was normalized [34] to cyclophilin (QuantiTect assay QT00177394) [21], which did not differ in transcript level between groups of offspring.

To determine the effect of Pe stimulation on Fads mRNA expression, total RNA was extracted from sections of aortae from male rats which were either untreated or treated with Pe (10^−4^ M) for 10 minutes, an equivalent exposure to that which induced maximum vasoconstriction and snap-frozen in liquid nitrogen. *Fads1* and *Fads2* mRNA expression was measured as described above.

### Analysis of rat *Fads1* and *Fads2* promoter methylation

The methylation status of individual CpG dinucleotides in the *Fads1* and *Fads2* promoters was measured by pyrosequencing, essentially as described [35]. Genomic DNA was isolated and bisulphite conversion was carried out using the EZ DNA methylation kit (ZymoResearch) [35]. Modified DNA was amplified using hot startTaq DNA polymerase (Qiagen) by PCR using the primers listed in Table S2. PCR products were immobilised on streptavidin–sepharose beads (Amersham), washed, denatured and released into annealing buffer containing sequencing primers (Table S2). Pyrosequencing was carried out using the SQA kit on a PSQ 96MA machine (Biotage) and the percentage methylation was calculated using the Pyro Q CpG programme (Biotage).

### Measurement of rat *Fads2* promoter activity

Promoter constructs containing wild type or CpG −394 C to A mutant *Fads2* promoters linked to a luciferase reporter gene were prepared essentially as described [36]. The *Fads2* promoter (−1038 bp to +318 bp) was amplified from rat genomic DNA by PCR using the primers 5′- ATCTCGAGCTCTGGTTCTTTTTCCTGGTA-3′ and 5′- ATAAGCTTAGAGGATGCTGCTAACTATCACCC-3′ and *Pfu* Proof reading Taq polymerase (Promega, Southampton, UK) [36]. Cycling conditions for PCR were 95°C for 2 minutes, then 40 cycles of 95°C for 1 minute, 58.9°C for 30 seconds and 72°C for 90 seconds. The resulting PCR product of 1356 bp were gel extracted, cloned into the pGL3 Basic reporter vector (Promega, Southampton, Hampshire, UK) and sequenced to confirm the presence of the insert. Mutagenesis was carried out using the QuikChange method (Stratagene, Texas, USA) to create a *Fads2* promoter construct with a mutated CpG −394 site [36]. Two complementary primers were designed using the QuikChange® Primer Design Program (Stratagene) to mutate the wild type *Fads2* sequence so that the cytosine at position −394 was mutated to an adenine. The primers used were 5′- TGAGTCTTATCTCTTCACTGATAATTTAGACCTTGTGGAAAAGG-3′ and 5′ CCTTTTCCACAAGGTCTAAATTATCAGTGAAGAGATAAGACTCA -3′. Primers (0.4 µM) were annealed to 30 ng P2- pGL3 DNA and extended using SequalPrep polymerase (Invitrogen, Paisley, UK). Cycling conditions for PCR were 94°C for 2 minutes, 12 cycles of 94°C for 10 seconds, 55°C for 30 seconds and 68°C for 1 minute, followed by 72°C for 5 minutes. The PCR products were digested with Dpn 1 at 37°C for 1 hour and the product transformed into DH5α E. Coli. Clones were cut with XhoI and HindIII, and then sequenced to confirm the presence of the mutation.

Rat hepatocarcinoma CC-1 cells were transfected with the wild type or mutated forms of the *Fads2* promoter using fuGENE HD Transfection Reagent (Promega) according to the manufacturer's instructions. Briefly, 2 µg of wild type and mutated *Fads2* promoter constructs and 2 µg of pRL-TK (internal Renilla luciferase control) were mixed with 12 µl of transfection reagent, incubated for 30 minutes at RT and then added drop wise to cells. After incubation at room temperature for 15 minutes, cells were washed and then incubated at 37°C, 5% CO_2_ for 4 hours. Because CpG −394 is located within a putative estrogen receptor response element, 17α-ethenylestradiol was used as a transcription agonist. Cells were then treated with 0, 70 and 700 pM 17α-ethenylestradiol for 24 hours, then washed with PBS and promoter activity was measured using the Dual-Luciferase Reporter Assay kit (Promega) on a TD-20/20 Turner Designs Luminometer (Turner Designs, CA, USA).

### Rat Aorta Eicosanoid Biosynthesis

Sections of aortae or mesenteric arteries were placed in tissue culture dishes containing physiological saline solution with or without SC26196 (100 µM) or sesamin (100 µM) for 30 minutes prior to treatment with Pe (1 µM) for 10 minutes. The supernatant was isolated and snap frozen in liquid nitrogen. The volume of supernatant recovered and the tissue mass were recorded. Eicosanoids were extracted from culture supernatants as described [37], [38]. Briefly, culture supernatants were adjusted to 15% methanol (v/v), and PGE_2_-*d*4 and 12-HETE-*d*8 (20 ng) added as internal standards [37], [38]. The samples were adjusted to pH 3.0 and applied to C18-E (500 mg) solid phase extraction cartridges (Phenomenex, Macclesfield, UK) which had been preconditioned with methanol and water. The cartridges were washed with 15% (v/v) methanol, water and hexane, and eicosanoids eluted by methyl formate. The organic solvent was evaporated and the remaining residue then reconstituted in ethanol (100 µl). Eicosanoids were separated on a C_18_ reversed-phase (RP) LC column (Phenomenex Luna, 3 µm particles, 150×2 mm) using a linear mobile phase gradient (A, 0.02% glacial acetic acid in water; B, 0.02% glacial acetic acid in acetonitrile) at 0.7 mL/min. Eicosanoids were identified and quantified using LTQ Velos linear ion trap (LIT-orbitrap) and QTRAP 4000 mass spectrometers [37], [38].

### Statistical Analysis

Dietary groups were compared by ANOVA with sex, total maternal dietary fat and fat type as between subject factors using Tukey's *post hoc* test (SPSS, Chicago, Illinois, USA). Vascular response to varying concentrations of vaso-relaxing and vasoconstricting agents were assessed by ANOVA with sex, total maternal dietary fat and fat type as between subject factors, and dose of drug as a within subject factor with Tukey's *post hoc* analysis. Statistical associations between mRNA expression and the level of methylation of individual CpG dinucleotides were assessed by calculation of Pearson's correlation coefficient. The investigators were blinded to the dietary grouping at all points during the experiment.

## Results

### Amount and Type of Maternal Dietary Fat Alters Acetylcholine-Induced Vaso-Relaxation in Offspring Aortae and Mesenteric Arteries

**Figure 1:**
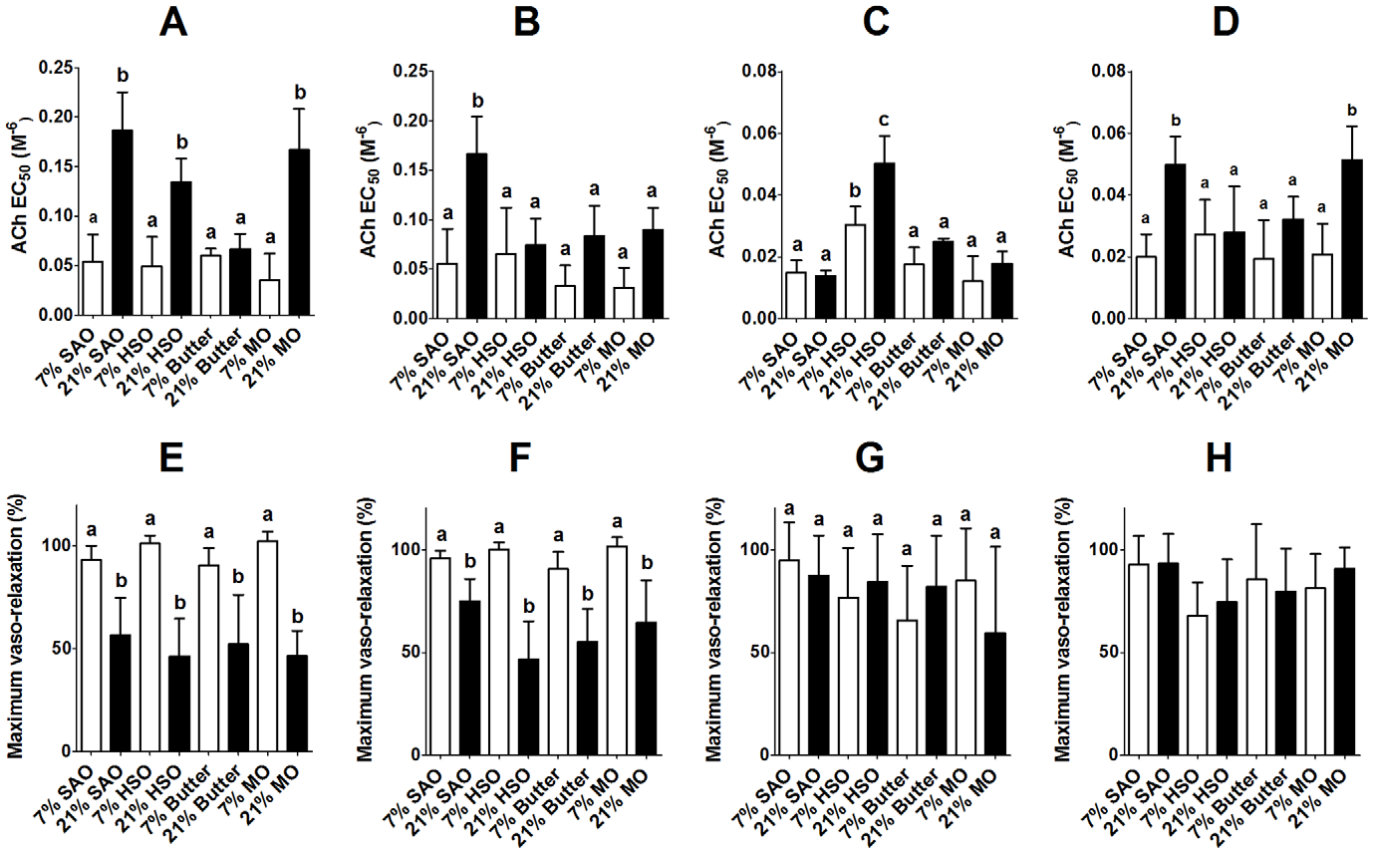
Amount and type of maternal dietary fat induced altered ACh-induced vaso-relaxation in the offspring. (A)–(D), Values are mean ± SD (n = 6/group) concentration which produced 50% maximum response (EC_50_) such that increasing values on the y-axis correspond to decreasing vaso-relaxation responses. (E)–(H), Values are mean ± SD (n = 6/group) maximum vaso-relaxation. (A,E) male and (B,F) female aortae, (C,G) male and (D,H) female mesenteric arteries. Statistical comparisons were by ANOVA with Tukey's *post hoc* analysis. Values significantly different (P<0.05) between maternal dietary groups within a sex are indicated by different letters.

The amount (P<0.0001) and type (P<0.0001) of maternal dietary fat induced altered ACh-mediated vaso-relaxation in offspring aortae, contingent on sex (P = 0.009; interaction sex*fat type*fat amount P = 0.013). Maternal diets containing 21% SAO, HSO and MO, but not butter, decreased ACh-mediated vaso-relaxation, indicated by increased EC_50_, in male offspring compared to offspring of dams fed diets containing 7% fat (Figure 1A). In female aortae, the response to ACh was only altered in the offspring of dams fed 21% SAO compared to the other dietary groups (Figure 1B).

There were significant effects of sex (P = 0.023) and amount (P<0.0001), but not type, of maternal dietary fat (interaction sex*fat amount P = 0.003) on ACh-mediated vaso-relaxation of mesenteric arteries. In males, the pattern of effects of maternal fat intake on the response of mesenteric arteries to ACh differed from those induced in the aorta. Male offspring of dams fed 7% HSO showed impaired vaso-relaxation compared to offspring of dams fed the other 7% diets, which was impaired further in offspring of dams fed 21% HSO compared to all other maternal dietary groups (Figure 1C). Female offspring of dams fed 21% fat showed a similar pattern of responses to ACh in mesenteric arteries compared to offspring of dams fed 7% to those in the aorta (Figure 1D).

There was a significant effect of the amount of maternal dietary fat (P<0.0001), but not sex or type of maternal dietary fat on the maximum ACh-induced vaso-relaxation in aortae (Figure 1 E,F). There was no significant effect of maternal diet or offspring sex on maximum vaso-relaxation in mesenteric arteries (Figure 1 G,H).

### Maternal Dietary Fat Does Not Alter Offspring *eNOS* mRNA Expression

To investigate whether maternal dietary fat altered vaso-relaxation in the offspring by altering the regulation of eNOS transcription, we measured the mRNA expression of *eNOS* in aortae. There were no significant differences in *eNOS* mRNA expression between male or female offspring of dams fed different amounts and types of fat (Figure S1 A,B).

### Amount of Maternal Dietary Fat Alters Offspring Phenylephrine-Mediated Vasoconstriction

**Figure 2:**
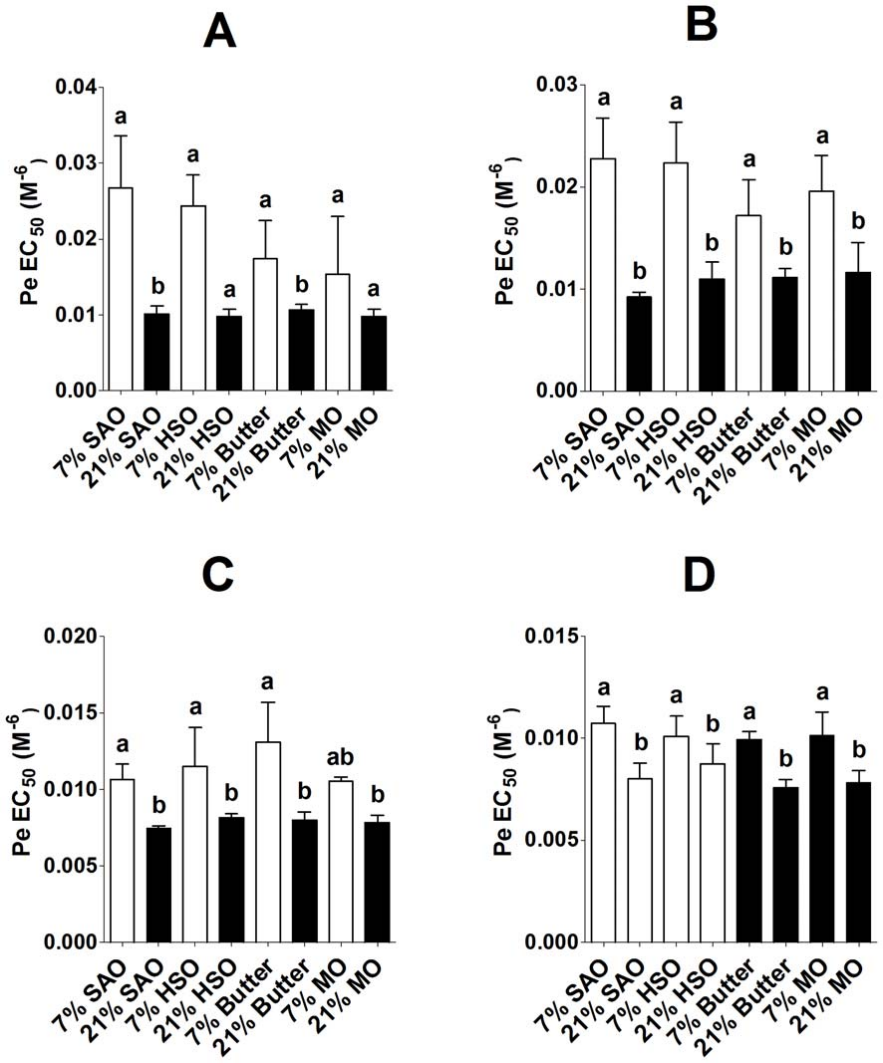
Amount and type of maternal dietary fat induced altered Pe-induced vasoconstriction in the offspring. Values are mean ± SD (n = 6/group) concentration which produced 50% maximum response (EC_50_) such that increasing values on the y-axis correspond to decreasing vasoconstriction. Statistical comparisons were by ANOVA with Tukey's *post hoc* analysis. Values significantly different (P<0.05) between maternal dietary groups within a sex are indicated by different letters. (A) Male and (B) female aortae, (C) male and (D) female mesenteric arteries.

There was a significant single factor effect of the amount (P<0.0001) of maternal dietary fat, but not type of dietary fat or of sex, on Pe-mediated vasoconstriction in offspring aorta (fat amount*sex interaction P<0.0001). There was a significant single factor effect of the amount of maternal dietary fat (P<0.0001), but not type of dietary fat or of sex, on Pe-mediated vasoconstriction in offspring mesenteric arteries. Male offspring of dams fed 21% fat showed increased vasoconstriction in response to Pe, indicated by lower EC_50_, in aorta and mesenteric arteries than male offspring of dams fed 7% fat (Figure 2 A,B). Similarly, female offspring of dams fed 21% fat showed increased vasoconstriction in response to Pe stimulation in aorta and mesenteric arteries than female offspring of dams fed 7% fat (Figure 2 C,D).

### Maternal Dietary Fat Alters Offspring Aorta and Plasma Fatty Acid Composition

**Figure 3:**
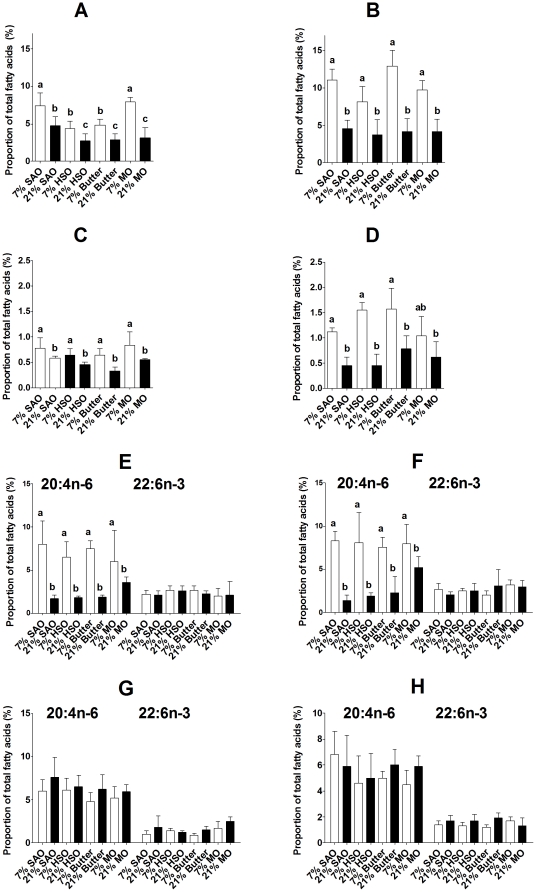
Amount and type of maternal dietary fat altered offspring aorta and plasma TAG PUFA composition. Values are mean ± SD (n = 6/group). Statistical comparisons were by ANOVA with Tukey's *post hoc* analysis. Values significantly different (P<0.05) within a sex are indicated by different letters. (A) Male and (B) female aorta 20:4n-6 content. (C) Male and (D) female aorta 22:6n-3 content. (E) Male and (F) female plasma triacylglycerol 20:4n-6 and 22:6n-3 contents. (G) Male and (H) female plasma non-esterified 20:4n-6 and 22:6n-3 contents.

There were small, but significant, differences in the proportion of 18:3n-3 in offspring aorta total lipids between maternal dietary groups (Table S3). The major changes in aorta fatty acid composition induced by maternal fat intake were in the proportions of 20:4n-6 and 22:6n-6 (Figure 3). There were no significant differences between groups in the proportions of the other PUFA measured (Table S3). Furthermore, TFA were not detected in aortae from 7% or 21% HSO offspring, nor in aortae from any of the other maternal dietary groups. There were significant effects on the proportion of 20:4n-6 in aorta total lipids of type (P = 0.007) and amount of maternal dietary fat (P<0.0001), and of sex (P = 0.0014) (interactions sex*fat type P = 0.002; fat amount*fat type P = 0.036). In males, the proportion of 20:4n-6 was lower in offspring of dams fed 7% HSO or 7% butter offspring compared to those of dams fed 7% SAO and MO (Figure 3A). There was no significant difference in the proportion of 20:4n-6 in aorta lipids between female offspring of the dams fed 7% fat (Figure 3B). Male and female offspring of dams fed 21% fat had a lower proportion of 20:4n-6 in aorta lipids irrespective of the type of fat (Figure 3 C,D). There were significant effects of the type (P = 0.02) and amount (P = 0.0004) of maternal dietary fat, and of offspring sex (P = 0.003) on the proportion of 22:6n-3 in offspring aortae (interactions sex*fat amount P = 0.001; sex*fat type P = 0.007; fat amount*fat type P = 0.04). The proportion of 22:6n-3 was lower in male and female offspring of dams fed 21% fat than offspring of those fed 7% fat (Figure 3 C,D).

In order to investigate whether the differences in aorta fatty acid composition between maternal dietary groups were related to plasma fatty acid composition, we measured the composition of plasma triacylglycerol (TAG) and non-esterified fatty acids (NEFA) which are the major transport pools that supply PUFA to blood vessels. There was a significant effect of the amount (P<0.0001), but not of fat type or of sex, on the proportion of 20:4n-6 in plasma TAG. The proportion of 20:4n-6 was lower in plasma TAG of male and female offspring of dams fed 21% fat compared to those of dams fed 7% fat (Figure 3 E,F). There was no significant difference between offspring of the different maternal dietary groups in the proportion of 22:6n-3 in plasma TAG (Figure 3 E,F), or in the proportions of 20:4n-6 or 22:6n-3 in plasma NEFA between dietary groups (Figure 3 G,H).

### Maternal Dietary Fat Alters Offspring Aorta *Fads 1* and *2* mRNA Expression

**Figure 4:**
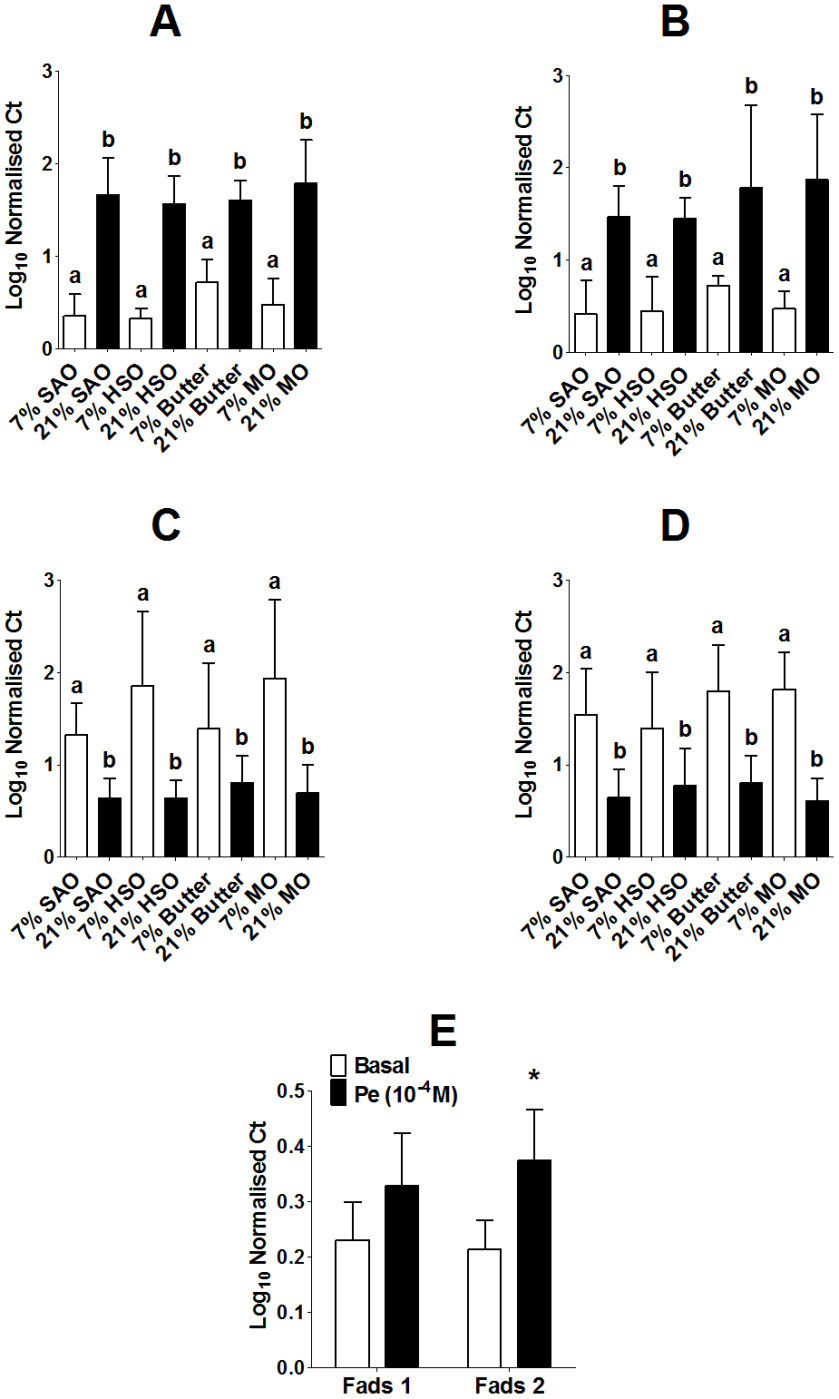
Amount and type of maternal dietary fat induced altered offspring *Fads1* and *Fads2* mRNA expression. Values are mean ± SD (n = 6/group). Statistical comparisons were by ANOVA with Tukey's *post hoc* analysis. Values significantly different (P<0.05) between maternal dietary groups within a sex indicated by different letters. (A) Male and (B) female *Fads1* expression, (C) Male and (D) female *Fads2* expression. (E) Fads1 and Fads 2 mRNA expression in untreated (basal) or stimulated with phenylephrine in aortae from adult male rats (n = 6). *Values significantly different from basal (P<0.05) by Student's unpaired t test.

The differences in aorta 20:4n-6 and 22:6n-3 composition between maternal dietary groups could not be explained simply by the proportions of these fatty acids in plasma TAG or NEFA. Therefore, we investigated whether differences in maternal dietary fat intake altered the capacity of the aorta to synthesise PUFA by measuring the mRNA expression of *Fads1* and *Fads2*. There was a significant effect of sex (P<0.0001) and of total maternal fat intake (P<0.0001), but not type of maternal dietary fat (interaction sex*fat amount P<0.0001) on *Fads1* mRNA expression. *Fads1* mRNA expression was significantly higher in both male and female offspring of dams fed 21% fat compared to those of dams fed 7% fat (Figure 4 A, B). There was a significant effect of the amount maternal dietary fat (P<0.0001), but not of fat type or of sex, on *Fads2* expression (Figure 4). *Fads2* mRNA expression was significantly lower in aorta of male and female offspring of dams fed the 21% fat diets compared to those of dams fed the 7% diets (Figure 4 C,D).

There was a non-significant trend (Student's unpaired t test; P<0.1) towards higher *Fads1* mRNA expression in sections of aortae stimulated with Pe than untreated aortae (Figure 4E). *Fads2* mRNA expression was 76% higher in Pe-treated than untreated aortae (P = 0.013).

### Inhibition of Δ6 and Δ5 Desaturases Impairs Ex-Vivo Vasoconstriction

**Figure 5:**
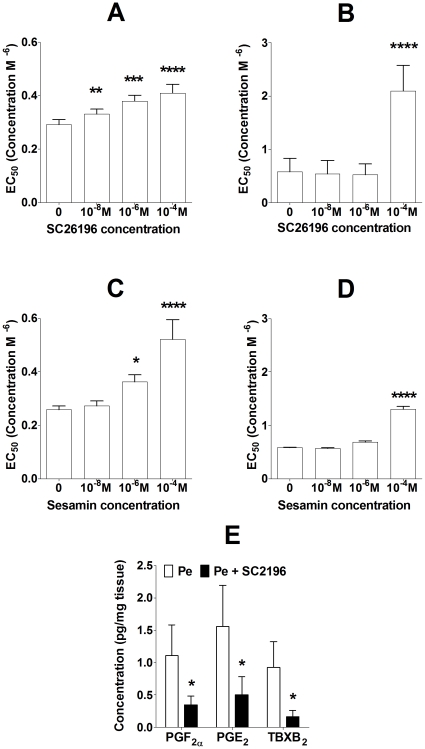
Δ6 and Δ5 desaturase activities are involved in Pe-mediated vasoconstriction. Values are mean ± SD (n = 6). (A) Pe+/−SC26196 in aortae; (B) Pe+/−SC26196 in mesenteric arteries; (C) Pe+/−sesamin in aortae; (D) Pe+/−sesamin in mesenteric arteries from male rats. Statistical comparisons of effect of dose of inhibitor were by 1-Way ANOVA with Dunnett's *post hoc* test; all ANOVA P<0.0001. E) Concentrations of pro-constriction eicosanoids synthesised from AA following Pe-stimulation of aorta+/−SC26196 (10^−4^ M) (n = 3 per treatment) from male rats. Comparisons of eicosanoid concentrations were by Student's paired test. Values significantly different from Pe treatment alone are indicated by *P<0.05, **P<0.01, ***P<0.001, ****, P<0.0001.

There was no significant effect of SC-26196 (Figure S2 A,B) or sesamin (data not shown) on ACh-mediated vaso-relaxation in aortae or mesenteric arteries. Treatment of aortae and mesenteric arteries with SC-26196 induced dose-related inhibition of Pe-mediated vasoconstriction in aortae, although inhibition of Pe-mediated vasoconstriction was only found at 0.1 mM SC-26196 in mesenteric arteries (Figure 5 A,B). Sesamin also induced a dose-related inhibition of Pe-mediated vasoconstriction in aortae, although in mesenteric arteries an effect was only detected at the highest sesamin concentration (Figure 5 C,D).

In order to investigate the mechanism by which SC-26196 inhibited Pe-mediated vasoconstriction, we measured in aortae the secretion of eicosanoids derived from 20:4n-6 in response to Pe stimulation. The concentrations of the pro-vasoconstriction eicosanoids PGF_2α_, PGE_2_ and TBXA_2_ (measured as its spontaneous degradation product TBXB_2_) were significantly lower in culture supernatants in the presence of SC26196 than when treated with Pe alone (Figure 5 E).

**Figure 6:**
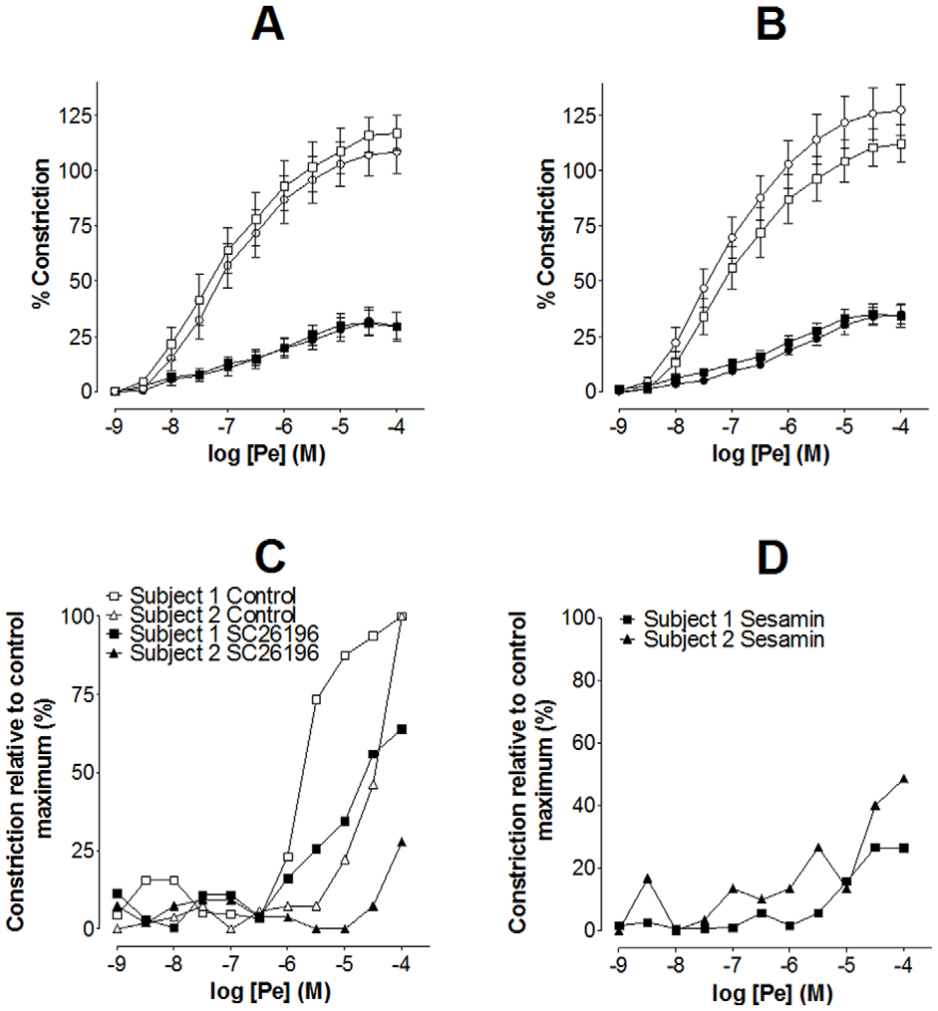
Δ6 and Δ5 desaturases are active in rat aorta smooth muscle and are involved in human femoral artery vasoconstriction. (A) and (B), rat aorta. Removal of the endothelium from male rat aorta did not alter inhibition of Pe-induced vasoconstriction by (A) Δ6 desaturase inhibitor SC26196 (10^−4^ M) or (B) Δ5 desaturase inhibitor sesamin (10^−4^ M). Values are mean ± SD (n = 6/treatment). Open circles, response to Pe in aorta with endothelium; open squares, response to Pe in aorta without endothelium; closed circles, response to Pe in aorta with endothelium treated with desaturase inhibitor; closed squares, response to Pe in aorta without endothelium treated with desaturase inhibitor. (A) Δ6 desaturase inhibitor SC26196 (10^−4^ M); Δ5 desaturase inhibitor sesamin (10^−4^ M). (C) and (D), human femoral artery. Treatment with (C) SC26196 (10^−4^ M) or (D) sesamin (10^−4^ M) decreased Pe-induced vasoconstriction in human femoral arteries collected from two subjects. Values are per cent constriction relative to maximum Pe-induced constriction. Control curves for panel D are shown in panel (D). Open symbols, Pe treatment alone; closed symbols, Pe treatment in the presence of desaturase inhibitor.

As expected, removal of the aortic endothelium tended to increase Pe-mediated vasoconstriction compared to intact vessels (Figure 6) [29] and abolished ACh-induced vaso-relaxation (data not shown). There was no significant effect of removing the endothelium on the inhibition of Pe-mediated vasoconstriction by SC26196 (Figure 6A) or sesamin (Figure 6B).

In order to determine whether PUFA biosynthesis is involved in vasoconstriction in human arteries, the effect of desaturase inhibition on Pe-induced vasoconstriction was measured in femoral arteries from two subjects. Despite differences between individuals in the magnitude of response to differing doses of Pe, treatment with SC26196 or sesamin reduced vasoconstriction in femoral arteries from both subjects (Figure 6 C,D).

### Maternal Dietary Fat Alters Methylation of the *Fads2* Promoter

**Figure 7:**
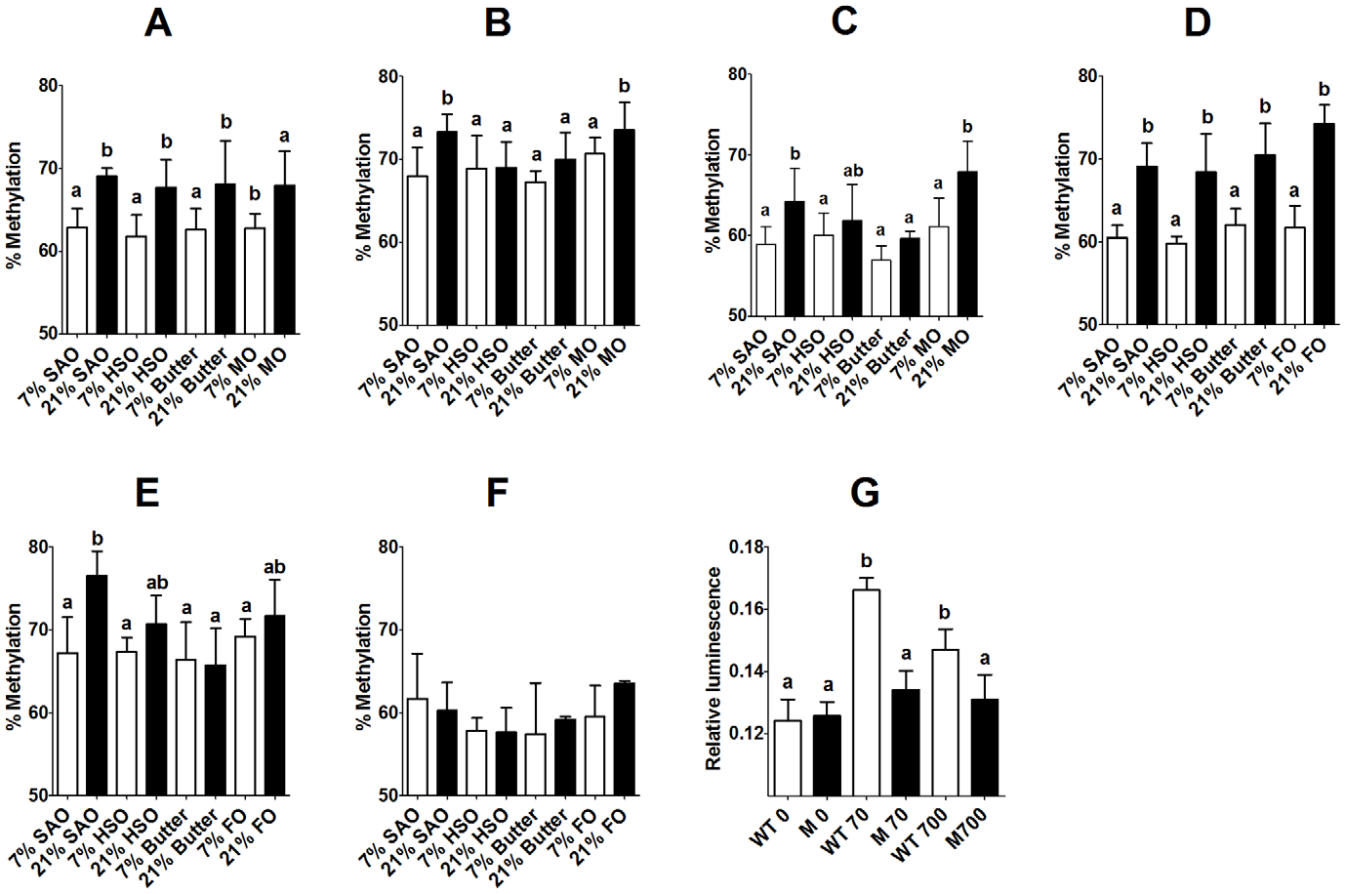
Amount and type of maternal dietary fat induced altered *Fads2* promoter methylation. Values are mean ± SD (n = 6/group). Statistical comparisons were by ANOVA with Tukey's *post hoc* analysis. Values significantly different (P<0.05) between groups re indicated by different letters. CpG dinucleotides are numbered by their location relative to the transcription start site (bp) (Online Figure 2). Male offspring; (A) CpG −394, (B) CpG −84, (C) CpG −76. Female offspring; (D) CpG −394, (E) CpG −84, (F) CpG −76. (G) Effect of mutation cytosine −394 to adenine on *Fads*2 promoter activity in response to estrogen. Values are mean ± SD for n = 6 replicate assays. Statistical analysis was by 1-Way ANOVA with Dunnett's *post hoc* test. ANOVA P = 0.003. Wild type (WT) promoter, M = mutated promoter.

Eleven CpGs were identified in a 5′-regulatory region between 0 and 800 bp from the *Fads2* transcription start site (Figure S3). Of these, only CpGs at −394, −84 and −76 bp from the transcription start site showed significant variation in methylation between maternal dietary groups, while the remainder did not show any significant differences between groups (Figure S4). There were significant single factor effects of amount of maternal dietary fat on the methylation of CpG −394 (P<0.0001), CpG −84 (P = 0.001) and CpG −76 (P = 0.036), and of the type of maternal dietary fat on CpG −84 (P = 0.009) and CpG −76 (P = 0.045). There were no significant differences between sexes in the methylation of any of the CpGs measured. CpG −394 was hypermethylated in male and female offspring of dams fed 21% fat diets compared to those fed 7% fat irrespective of fat type (Figure 7 A,D). CpG −84 was hypermethylated in offspring of dams fed 21% SAO or 21% MO compared to the equivalent 7% diets in males, but was only altered significantly in female offspring of 21% SAO dams (Figure 7 B,E). CpG −76 was hypermethylated in offspring of dams fed 21% SAO, HSO and MO compared those fed the equivalent 7% fat diets in male offspring, but there were no statistically significant differences in the methylation of CpG −76 in female offspring (Figure 7 C,F). Methylation of CpG −394, but not CpG −76 or CpG −84, which is located within a putative estrogen receptor response element (Figure S3), was negatively correlated with *Fads2* expression in male (r = −0.373, p = 0.018) and female (r = 0.366, P = 0.031) offspring.

Eleven CpG dinucleotides were detected in CpG rich a 5′-regulatory region between 0 and 120 bp from the *Fads1* transcription start site (Figure S5). There were no significant differences between maternal dietary groups in the methylation of any of the CpG dinucleotides measured in the *Fads1* promoter in male or female offspring (Figure S6).

We confirmed the role of CpG −394 in regulating *Fads2* expression by mutating cytosine to adenine at position −394. Because CpG −394 is located in a putative estrogen receptor response element (Figure S3), we measured the change in transcription of the cloned wild type and mutated promoters in response to stimulation with estrogen. There was a significant dose-related increase in the activity of the wild type *Fads2* promoter in response to estrogen treatment, which was abolished in the mutated promoter (Figure 7G).

## Discussion

Our findings show that both the amount and type of maternal dietary fat consumed during pregnancy and lactation induced persistent changes in both vaso-relaxation and vasoconstriction in the offspring, although the amount of fat in the maternal diet appears to have the dominant effect. We demonstrate for the first time that 20:4n-6 biosynthesis *de novo* in arterial smooth muscle is required for Pe-induced vasoconstriction, and that increased maternal fat intake induced dysregulation of *Fads1* and *Fads2* transcription in offspring aortae which involves altered epigenetic regulation of the *Fads2* promoter.

Previous studies have shown that feeding either a high saturated fat or n-3 PUFA-deficient diet during pregnancy and lactation induces altered blood pressure and endothelial function in the offspring [17]–[20]. The present findings show, for the first time, that consuming different types and amounts of fat during pregnancy and lactation in rats induced differences in the regulation of vascular tone in the offspring which resembled human endothelial dysfunction in CVD [1], [2]. Such effects differed between sexes, and between conduit and resistance arteries. Furthermore, increasing the amount of maternal dietary fat did not simply exacerbate the changes in vascular tone induced by different types of maternal dietary fat. For example, aortae of male offspring of dams fed 21% SAO reduced response to ACh than offspring of dams fed 7% SAO, while there was no difference in the response to ACh between male offspring of dams fed 7% or 21% butter. Together these findings suggest specificity in the response of the developing vascular system to fatty acids, although the precise mechanism underlying such effects cannot be deduced from the present study. The magnitude of the difference in response to ACh and Pe between the offspring of dams fed the 21% and 7% fat diets was comparable to that associated with an approximate difference of 50% in radial artery flow-mediated dilatation in humans [39]. This suggests that such ex-vivo measurements are of direct relevance to vascular function *in vivo*.

The 21% fat maternal diets decreased sensitivity to ACh and decreased the maximum level of vaso-relaxation in offspring aortae with some contingency for sex and type of maternal fat. There was less effect on these parameters in mesenteric arteries. ACh-induced vaso-relaxation in aortae is dependent primarily on NO, while in mesenteric arteries is due to the activities of both NO and endothelium-derived hyperpolarising factor (EDHF) [40]. Maternal protein restriction during pregnancy in rats has been shown to reduce NO activity and eNOS mRNA expression [41]. This suggests that regulation of NO biosynthesis is sensitive to the early life environment. The greater effect of maternal dietary fat on aortae compared with mesenteric arteries is consistent with this suggestion. Furthermore, EDHF has been shown to compensate for decreased NO activity and/or bioavailability [42] and so the blunted effect of maternal dietary fat on mesenteric arteries may reflect, at least in part, compensation by EDHF for any effect on NO.

There was no effect of maternal fat intake on eNOS mRNA expression and so altered epigenetic regulation of NO synthesis is unlikely to be involved in altered response to ACh induced in the offspring by maternal dietary fat, although it is possible that changes in the transcriptional regulation of other enzymes involved in NO biosynthesis may be involved. eNOS activity is modulated by the fatty acid composition of cell membranes and of caveolae [43], [44]. Thus induced changes in arterial membrane fatty acid composition may alter eNOS activity by changing the biophysical properties of arterial cell membranes and so contribute to differences in vaso-relaxation between maternal dietary groups. One previous report has shown that feeding a high SFA diet induced lower proportions of 20:4n-6 and 22:6n-3 in rat aorta [45]. Our findings are in agreement with this study [45], but extend it by showing that high maternal fat intake decreased the proportion of 20:4n-6 and 22:6n-3 in aorta of the offspring irrespective of the type of maternal dietary fat. Such changes in the fatty acid composition of blood vessels may alter eNOS activity and NO synthesis. However, since there were no differences in the proportions of 20:4n-6 and 22:6n-3 between different types of maternal dietary fat, other factors in addition to altered membrane composition are likely to be involved in the changes in vaso-relaxation induced in the offspring.

Vasoconstriction involves the activities of specific eicosanoids including PGE_2_, PGF_2α_ and TBXA_2_. Synthesis of vasoactive eicosanoids involves release of 20:4n-6 from membrane phospholipids by cytosolic phospholipase A_2_ (cPLA_2_) [46], followed by cyclooxygenase activity [47] leading to the formation of unstable PGH_2_ which is subsequently converted to individual eicosanoid species by the activities of specific prostanoid synthases [48]. Therefore, a lower proportion of 20:4n-6 in arterial membranes would be expected to decrease the synthesis of pro-constrictor eicosanoids. However, feeding the maternal 21% fat diets increased vasoconstriction in both aortae and mesenteric arteries irrespective of the type of fat or the sex of the offspring, which implies a mismatch between the availability of the 20:4n-6 substrate and capacity of the arteries to constrict. 20:4n-6 in arterial cell membranes may be derived either from dietary 20:4n-6 or 20:4n-6 synthesised from 18:2n-6 by the liver and transported in plasma lipid pools, in particular TAG and NEFA. 20:4n-6 may also be synthesised *de novo* within the arterial cells. The pattern of induced changes in aortae fatty acid composition did not match the composition of the maternal diets. This suggests that differences in the fatty acid composition of arteries in the offspring were not due to persistence of fatty acids derived from the maternal diet, but rather reflected changes in the regulation of fatty acid supply or of membrane composition. The proportion of 20:4n-6 was decreased in plasma TAG of offspring of dams fed the 21% fat diets, but there was no difference between dietary groups in the proportion of 22:6n-3 in plasma TAG or NEFA. Together these findings suggest that differences in aorta fatty acid composition between offspring of dams fed the 7% or 21% fat diets cannot be attributed to altered supply of fatty acid substrates from blood alone. Furthermore, differences in availability of 20:4n-6 from blood cannot explain increased capacity for vasoconstriction in the offspring of dams fed the 21% fat diets.

Synthesis of 20:4n-6 and 22:6n-3 by Δ6 and Δ5 desaturase activities has been shown previously in isolated vascular endothelial [13], [49] and smooth muscle [14] cells, although the function of this pathway in vascular tissues has not been described. We found that offspring of dams fed the 21% fat diets had higher expression of *Fads1* and lower expression of *Fads2* in aorta compared to offspring of dams fed the 7% fat diets, irrespective of the type of maternal dietary fat. To some extent, these findings are consistent with previous studies which have shown that TFA, which induce endothelial dysfunction in adult humans and animals models [50], reduce the proportions of 20:4n-6 and 22:6n-3 in blood in neonatal pigs [51], in human infants [52], [53] and in children [54]. Such effects have been suggested to be due to inhibition of hepatic Δ6 desaturase [55] leading to reduced supply of 20:4n-6 for uptake by blood vessels and, in turn, for eicosanoid synthesis [36]. Furthermore, an acute increase in fat intake inhibited Δ6 and Δ5 desaturase activities through down-regulation of the transcription of their genes *Fads2* and *Fads1* [56], [57]. However, the present findings suggest that in arteries, maternal high fat feeding induces dysregulation of *Fads* expression in a manner that predicts increased synthesis of 20:4n-6 due to increased *Fads1* expression which is consistent with increased Pe-mediated vasoconstriction, but because of down-regulation of *Fads 2* expression also implies dysregulation of PUFA metabolism. Thus one implication of these findings is that synthesis of 20:4n-6 in artery walls is involved in vasoconstriction. Because the proportions of 20:4n-6 and 22:6n-3 were reduced in aortae of the offspring of dams fed the 21% fat diets, these findings also suggest that PUFA synthesised *de novo* in arterial walls are channelled, at least in part, into eicosanoid biosynthesis rather than incorporated into cell membranes.

Since inhibition of Δ6 and Δ5 desaturase reduced vasoconstriction, but not vaso-relaxation, PUFA biosynthesis *de novo* appears to have a specific role in the regulation of α_1_-adrenergic receptor-mediated vasoconstriction. This is supported by the reduction in the synthesis of specific, 20:4n-6-derived pro-constriction eicosanoids, PGF_2α_, PGE_2_ and TBXA_2_, when aortae were treated with Pe in the presence of SC26196. These findings are, to some extent, analogous to acute induction of 20:4n-6 biosynthesis from 20:3n-6 for eicosanoid production in murine macrophages treated with zymosan [58] and in human lymphocytes stimulated with phorbol myristate acetate [59]. Furthermore, PUFA synthesis *de novo* also appears to be involved in α_1_-adrenergic receptor-mediated vasoconstriction in humans, albeit in samples collected from only two subjects, which is consistent with the demonstration of PUFA biosynthesis in isolated human coronary artery smooth muscle cells [14]. Such local PUFA biosynthesis may ensure adequate availability of substrates for the synthesis of lipid second messengers irrespective of fatty acid supply from the blood stream. Previous reports which have shown an association between polymorphisms in *Fads1* and *Fads2* and increased risk of CVD in humans have assumed that the underlying mechanism involves impaired hepatic PUFA biosynthesis and supply to blood vessels [10]. In particular, polymorphisms which decrease *Fads* expression and plasma 20:4n-6 concentration appear to be cardioprotective [60], while those which increase Fads expression and 20:4n-6 status are associated with increased risk of atherosclerotic disease [61]. These findings are in direct agreement with our observations, but the present data suggest an alternative explanation for the association between risk of CVD and *Fads* genotype in that this may be due to altered capacity for PUFA biosynthesis *de novo* in arterial walls leading to impaired local control of vascular tone rather than to altered supply of 20:4n-6 synthesised by the liver.

Previous studies have demonstrated PUFA biosynthesis in vascular endothelial [13], [49] and smooth muscle [14]. Our findings show that removal of the endothelium from rat aortae did not alter the inhibition of Pe-mediated vasoconstriction by either SC26196 or sesamin. This suggests that the PUFA biosynthesis pathway which is involved in arterial vasoconstriction is located in smooth muscle rather than endothelial cells. This implies that eicosanoids which are formed from 20:4n-6 synthesised *de novo* in smooth muscle cells may act on TP receptors via an autocrine or paracrine mechanism.

There is evidence that differences in maternal nutrition induce persistent changes in the transcription of specific genes in the offspring, leading to altered phenotypes, by a mechanism involving changes in their epigenetic regulation by DNA methylation [15]. Expression of *Fads2* has been shown to be regulated by the methylation of specific CpG dinucleotides in its promoter in mice [62]. CpG −394 in the *Fads2* gene was hypermethylated in offspring of dams fed the 21% fat diets and was negatively associated with the level of *Fads2* mRNA. Since mutation of CpG −394 showed that it is involved in the regulation of *Fads2* transcription, these findings suggest that lower levels of *Fads2* mRNA in the offspring of dams fed 21% fat compared to those of dams fed 7% fat were due to altered epigenetic regulation of its promoter. In contrast, there was no effect of maternal fat intake on the methylation of CpG dinucleotides in the *Fads1* promoter. However, one possible explanation for the present findings is that the increased level of *Fads1* mRNA may reflect over-compensation for the decrease in the transcriptional activity of *Fads2*.

Because control of Fads2 transcription appeared to be important in the regulation of PUFA biosynthesis, we measured the mRNA expression of *Fads1* and *Fads2* in aortae stimulated with PE for a period of time equivalent to that which induced maximum vasoconstriction. There was a trend towards higher Fads1 and a significant increase in Fads2 mRNA expression following PE stimulation. This suggests that *Fads2* may function as an immediate early gene in Pe-induced vasoconstriction. This is supported by the finding that Pe stimulates phosphorylation and activation of the immediate early gene regulator *Elk-1* [63] and the presence of an active *Elk-1* response elements in the human *FADS2* promoter [64]. Analysis of the rat *Fads2* promoter by Genomatix MatInspector (http://www.genomatix.de) identified eight putative *Elk-1* response elements within 18.8 kbp upstream of the transcription start site (data not shown) which suggests Elk-1 may be an important regulator of this gene. Together these findings imply that transcriptional regulation of *Fads2* may be important for control of vascular tone and so may have implications for risk of cardiovascular disease. One mechanism for this may be variations in epigenetic processes or polymorphisms that alter the response of *Fads2* transcription to stimuli and so modulate vasoconstriction.

The findings of this study show that the regulation of vascular tone in offspring can be modified by maternal fat intake during pregnancy. The effect involves altered epigenetic regulation of arterial PUFA biosynthesis *de novo*, which represents a novel pathway in the regulation of vasoconstriction, and changes in the proportions of specific PUFA in arterial cell membranes. One implication of these findings is that the quality as well as the quantity of maternal dietary fat may be an important factor in future risk of CVD in humans.

## Supporting Information

Figure S1 **Examples of the responses of male aortae to ACh and Pe and aortae** ***eNOS*** **mRNA expression.** (A) ACh-induced vaso-relaxation and (B) Pe-induced vasoconstriction in the aortae of male offspring of dams fed either 7% or 21% SAO. eNOS mRNA expression in (C) male and (D) female offspring aortae. Values are mean ± SD (n = 6/group). For eNOS expression, statistical comparisons were by ANOVA with Tukey's *post hoc* analysis. There were no statistically significant differences between groups.
(PDF)

Figure S2 **Δ6 and Δ5 desaturase activities are not involved in ACh-mediated vaso-relaxation.** Values are mean ± SD (n = 6). (A) ACh+/−SC26196 in aortae; (B) ACh+/−SC26196 in mesenteric arteries. Statistical comparisons of effect of dose of inhibitor were by 1-Way ANOVA with Dunnett's *post hoc* test. Values significantly different from ACh treatment alone are indicated by *P<0.05, **P<0.01, ***P<0.001, ****, P<0.0001.
(PDF)

Figure S3 **Sequence of the 5′ regulatory region of rat** ***Fads2*** **.** CpG dinucleotides are indicated by bold text. Numbers above the text indicate the locations relative to the transcription start site (bases) of individual CpG dinucleotides which were measured by pyrosequencing. Underlined text indicates the location of a putative estrogen receptor response element. Arrow marks the transcription start site.
(PDF)

Figure S4 **Methylation status of CpG dinucleotides in the** ***Fads2*** **promoter in male and female offspring aortae.** Values are mean ± SD (n = 6/group). Statistical comparisons were by ANOVA with Tukey's *post hoc* analysis. There were no statistically significant differences between groups for these CpG dinucleotides. CpG dinucleotides are identified as distance (bp) from the transcription start site.
(PDF)

Figure S5 Sequence of the 5′ regulatory region of rat *Fads1*. CpG dinucleotides are indicated by bold text. Numbers above the text indicate the locations relative to the transcription start site (bases) of individual CpG dinucleotides which were measured by pyrosequencing. Arrow marks the transcription start site.
(PDF)

Figure S6 **Methylation status of CpG dinucleotides in the** ***Fads1*** **promoter in male and female offspring aortae.** Values are mean ± SD (n = 6/group). Statistical comparisons were by ANOVA with Tukey's *post hoc* analysis. CpG dinucleotides are identified as distance (bp) from the transcription start site. There were no statistically significant differences between groups for these CpG dinucleotides.
(PDF)

Table S1 Composition of diets. SOA, safflower oil; HSO, hydrogenated soybean oil; MO, Menhaden oil. Total n-6 PUFA is the sum of 18:2n-6, 18:3n-6, 20:3n-6, 20:4n-6, 22:4n-6 and 22:5n-6. Total n-3 PUFA is the sum of 18:3n-3, 20:5n-3, 22:5n-3 and 22:6n-3. N = not detected.
(PDF)

Table S2 Pyrosequencing primers.
(PDF)

Table S3 Polyunsaturated fatty acid composition of offspring aorta total lipids on day 77. Values are mean ± SD proportions of fatty acid in individual maternal plasma lipid classes (n = 6 offspring per group). Different superscripts indicate values which were significantly different (P<0.05) by a general linear model with Tukey's *post hoc* analysis. The proportions of 20:4n-6 and 22:6n-3 are reported in Figure 3.
(PDF)
