# Supplementary material for: Vascular Dysfunction Induced in Offspring by Maternal Dietary Fat Involves Altered Arterial Polyunsaturated Fatty Acid Biosynthesis
Source: PLoS One. 2012 Apr 3;7(4):e34492. doi: 10.1371/journal.pone.0034492 (PMC3317992; doi:10.1371/journal.pone.0034492)
Supplement: Figure S1 — Examples of the responses of male aortae to ACh and Pe and aortae eNOS mRNA expression. (A) ACh-induced vaso-relaxation and (B) Pe-induced vasoconstriction in the aortae of male offspring of dams fed either 7% or 21% SAO. eNOS mRNA expression in (C) male and (D) female offspring aortae. Values are mean ± SD (n = 6/group). For eNOS expression, statistical comparisons were by ANOVA with Tukey's post hoc analysis. There were no statistically significant differences between groups. (PDF) [file pone.0034492.s001.pdf]

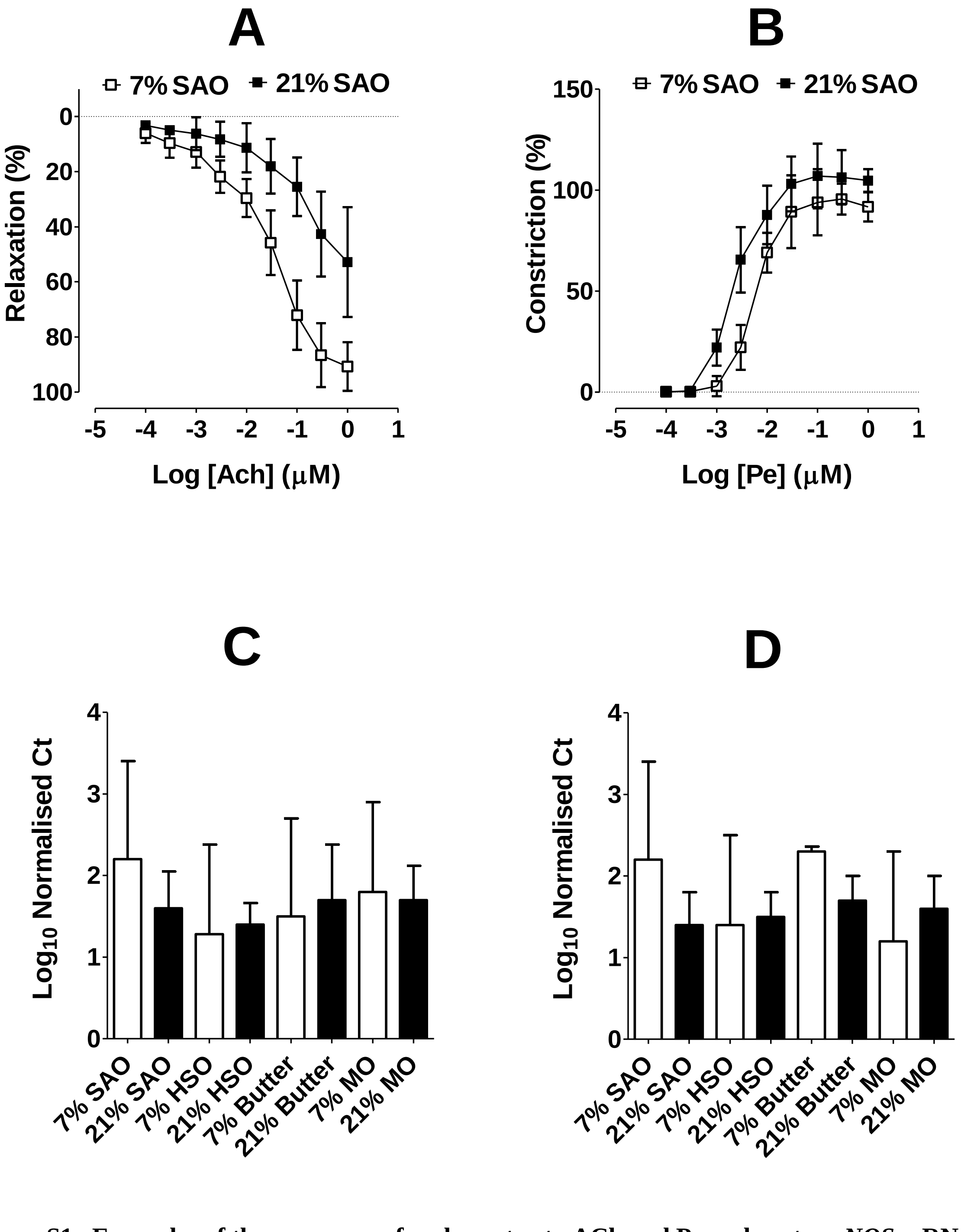

Figure S1. Examples of the responses of male aortae to ACh and Pe and aortae *eNOS* mRNA expression. (A) ACh-induced vaso-relaxation and (B) Pe-induced vasoconstriction in the aortae of male offspring of dams fed either 7% or 21% SAO. eNOS mRNA expression in (C) male and (D) female offspring aortae. Values are mean ± SD (n = 6 / group). For eNOS expression, statistical comparisons were by ANOVA with Tukey's *post hoc* analysis. There were no statistically significant differences between groups.
