# Supplementary material for: Vascular Dysfunction Induced in Offspring by Maternal Dietary Fat Involves Altered Arterial Polyunsaturated Fatty Acid Biosynthesis
Source: PLoS One. 2012 Apr 3;7(4):e34492. doi: 10.1371/journal.pone.0034492 (PMC3317992; doi:10.1371/journal.pone.0034492)
Supplement: Figure S2 — Δ6 and Δ5 desaturase activities are not involved in ACh-mediated vaso-relaxation. Values are mean ± SD (n = 6). (A) ACh+/−SC26196 in aortae; (B) ACh+/−SC26196 in mesenteric arteries. Statistical comparisons of effect of dose of inhibitor were by 1-Way ANOVA with Dunnett's post hoc test. Values significantly different from ACh treatment alone are indicated by *P<0.05, **P<0.01, ***P<0.001, ****, P<0.0001. (PDF) [file pone.0034492.s002.pdf]

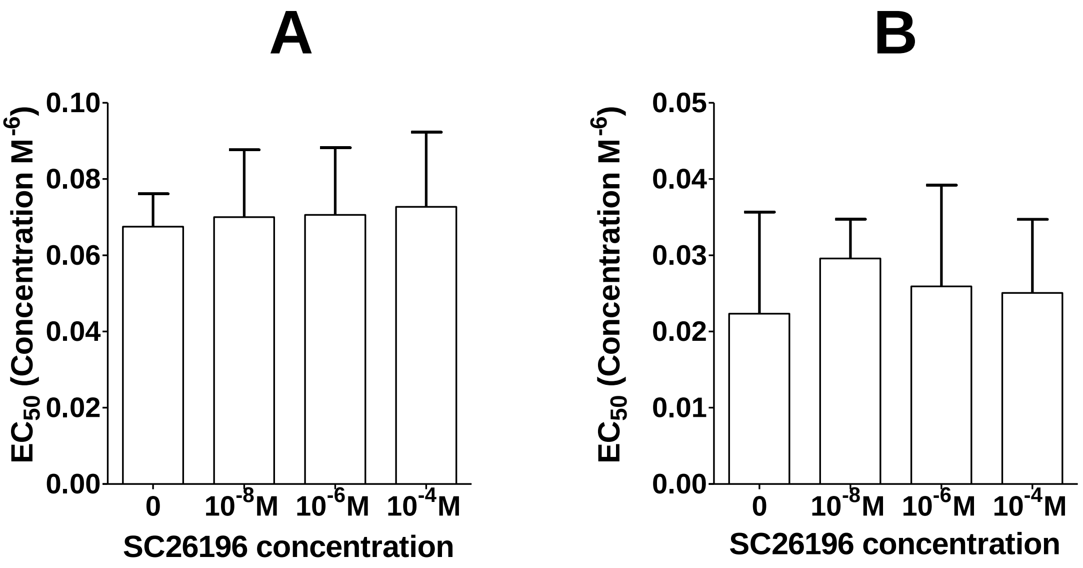

Figure S2. Δ6 and Δ5 desaturase activities are not involved in ACh-mediated vaso-relaxation. Values are mean ± SD (n = 6). (A) ACh +/- SC26196 in aortae; (B) ACh +/- SC26196 in mesenteric arteries. Statistical comparisons of effect of dose of inhibitor were by 1-Way ANOVA with Dunnett's *post hoc* test. Values significantly different from ACh treatment alone are indicated by *P<0.05, **P<0.01, ***P<0.001, ****, P<0.0001.
