# Supplementary material for: Vascular Dysfunction Induced in Offspring by Maternal Dietary Fat Involves Altered Arterial Polyunsaturated Fatty Acid Biosynthesis
Source: PLoS One. 2012 Apr 3;7(4):e34492. doi: 10.1371/journal.pone.0034492 (PMC3317992; doi:10.1371/journal.pone.0034492)
Supplement: Figure S6 — Methylation status of CpG dinucleotides in the Fads1 promoter in male and female offspring aortae. Values are mean ± SD (n = 6/group). Statistical comparisons were by ANOVA with Tukey's post hoc analysis. CpG dinucleotides are identified as distance (bp) from the transcription start site. There were no statistically significant differences between groups for these CpG dinucleotides. (PDF) [file pone.0034492.s006.pdf]

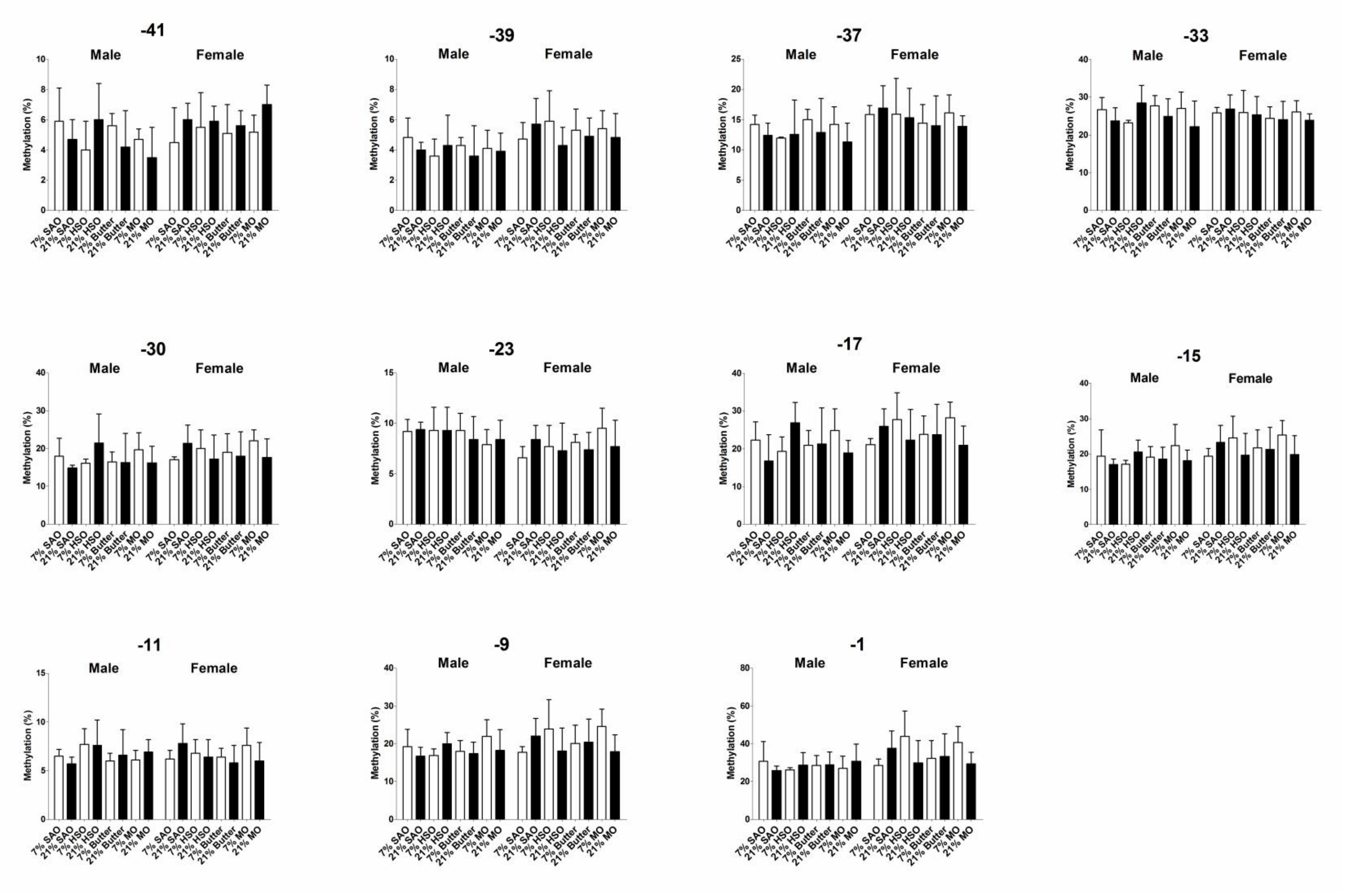

Figure S6. Methylation status of CpG dinucleotides in the *Fads1* promoter in male and female offspring aortae. Values are mean ± SD (n = 6 / group). Statistical comparisons were by ANOVA with Tukey's *post hoc* analysis. CpG dinucleotides are identified as distance (bp) from the transcription start site. There were no statistically significant differences between groups for these CpG dinucleotides.
