# Supplementary material for: Vascular Dysfunction Induced in Offspring by Maternal Dietary Fat Involves Altered Arterial Polyunsaturated Fatty Acid Biosynthesis
Source: PLoS One. 2012 Apr 3;7(4):e34492. doi: 10.1371/journal.pone.0034492 (PMC3317992; doi:10.1371/journal.pone.0034492)
Supplement: Table S1 — Composition of diets. SOA, safflower oil; HSO, hydrogenated soybean oil; MO, Menhaden oil. Total n-6 PUFA is the sum of 18:2n-6, 18:3n-6, 20:3n-6, 20:4n-6, 22:4n-6 and 22:5n-6. Total n-3 PUFA is the sum of 18:3n-3, 20:5n-3, 22:5n-3 and 22:6n-3. N = not detected. (PDF) [file pone.0034492.s007.pdf]

Table S1. Diet composition

	Maternal diets								Post-weaning diet
	7% (w/w) Fat				21% (w/w) fat				
	SAO	HSO	Butter	MO	SAO	HSO	Butter	MO	AIN93M
Casein (g/100g)	20.0	20.0	20.0	20.0	20.0	20.0	20.0	20.0	14.0
Corn starch (g/100g)	39.7	39.7	39.7	39.7	25.7	25.7	25.7	25.7	46.6
Sucrose (g/100g)	10.0	10.0	10.0	10.0	10.0	10.0	10.0	10.0	10.0
Maltodextrin (g/100g)	13.2	13.2	13.2	13.2	13.2	13.2	13.2	13.2	15.5
Cellulose (g/100g)	5.0	5.0	5.0	5.0	5.0	5.0	5.0	5.0	5.0
AIN93G mineral mix (g/100g)	3.5	3.5	3.5	3.5	3.5	3.5	3.5	3.5	3.5
AIN93G vitamin mix (g/100g)	1.0	1.0	1.0	1.0	1.0	1.0	1.0	1.0	1.0
Choline (g/100g)	0.25	0.25	0.25	0.25	0.25	0.25	0.25	0.25	0.25
α-Tocopheroyl acetate (mg/100g)	84	84	84	84	84	84	84	84	84
Total metabolisable energy (MJ/100g)	1.6	1.4	1.6	1.6	1.9	1.1	1.8	1.9	1.6
	Fatty acid composition (mg/100g)								
10:0	N	N	6	N	N	N	17	1	N
12:0	N	N	7	N	N	N	22	N	N
14:0	1	2	92	60	4	5	275	181	1
16:0	46	80	283	126	137	241	849	379	76
16:1n-7	1	N	12	75	2	1	35	226	1
18:0	19	89	88	24	57	268	264	73	29
18:1n-9	118	177	175	68	355	532	524	203	152
18:1n-7	4	20	7	25	13	60	20	74	10
Total TFA	N	305	N	N	N	914	N	N	N
18:2n-6	505	19	16	14	1515	58	49	42	376
18:3n-6	N	N	N	3	N	N	N	8	2
18:3n-3	1	2	4	8	2	5	13	23	48
20:0	2	3	4	2	7	9	13	5	2
22:1n-9	N	N	1	24	N	N	2	71	N
20:1n-9	1	N	N	9	4	N	N	27	1
20:2n-9	N	N	1	2	N	N	3	5	N
20:3n-6	N	N	1	2	N	N	2	6	N
20:4n-6	N	N	1	10	N	N	2	29	N
22:0	2	3	1	0	5	8	2	0	2
20:5n-3	N	N	1	142	N	N	3	427	N
24:0	N	N	N	N	N	N	N	0	N
22:4n-6	N	N	N	1	N	N	N	4	N
24:1n-9	N	N	N	2	N	N	N	7	N
22:5n-6	N	N	1	2	N	N	2	6	N
22:5n-3	N	N	1	19	N	N	4	58	N
22:6n-3	N	N	1	83	N	N	2	248	N
SFA	70	177	480	213	209	530	1441	640	110
MUFA	124	198	194	202	373	593	581	606	165
Total n-6 PUFA	505	19	17	29	1515	58	52	88	378
Total n-3 PUFA	1	2	7	252	2	5	22	755	48
18:2n-6 : 18:3n-3	739	13	4	2	739	13	4	2	8

SOA, safflower oil; HSO, hydrogenated soybean oil; MO, Menhaden oil. Total n-6 PUFA is the sum of 18:2n-6, 18:3n-6, 20:3n-6, 20:4n-6, 22:4n-6 and 22:5n-6. Total n-3 PUFA is the sum of 18:3n-3, 20:5n-3, 22:5n-3 and 22:6n-3. N = not detected.
