# Supplementary material for: Vascular Dysfunction Induced in Offspring by Maternal Dietary Fat Involves Altered Arterial Polyunsaturated Fatty Acid Biosynthesis
Source: PLoS One. 2012 Apr 3;7(4):e34492. doi: 10.1371/journal.pone.0034492 (PMC3317992; doi:10.1371/journal.pone.0034492)
Supplement: Table S2 — Pyrosequencing primers. (PDF) [file pone.0034492.s008.pdf]

Table S2. Pyrosequencing primers

Primer location (bp relative to transcription start site)	Forward (5' to 3')	Reverse (3' to 5')
	Fads1	
	PCR primers	
-107 to +179	GGTTTAGTTAATGGGAGGG	CCACCTCCTCCCAAATAAAATAA
	Sequencing primers	
-57	GGTGTTGAAAATTTTG	
	Fads2	
	PCR primers	
-766 to -473	TTGTGTATTTTTTGATGTGGTTAGATT	CCCAACTCAATCCCTAACACCTA
-499 to -318	TTTTAGGTGTTAGGGATTGAGT	ACTCCTAAACTAAATATCCTTCAATATACA
-139 to +165	TTGTTATTGAGGAGTGGGAAGTT	CCCCTACCATCCAACCTCAT
	Sequencing primers	
-748	GGTTAGATTTGAGTGTG	
-646	AAATGAAGTGTAATTTATGTTT	
-421	TGAGTTTTATTTTTTTATTGATAAT	
-112	ATTTGGTTTTTGAGTAGATA	
-49	GTTTTTTTGTTTTTTAGGATGTT	
