# Supplementary material for: Vascular Dysfunction Induced in Offspring by Maternal Dietary Fat Involves Altered Arterial Polyunsaturated Fatty Acid Biosynthesis
Source: PLoS One. 2012 Apr 3;7(4):e34492. doi: 10.1371/journal.pone.0034492 (PMC3317992; doi:10.1371/journal.pone.0034492)
Supplement: Table S3 — Polyunsaturated fatty acid composition of offspring aorta total lipids on day 77. Values are mean ± SD proportions of fatty acid in individual maternal plasma lipid classes (n = 6 offspring per group). Different superscripts indicate values which were significantly different (P<0.05) by a general linear model with Tukey's post hoc analysis. The proportions of 20:4n-6 and 22:6n-3 are reported in Figure 3. (PDF) [file pone.0034492.s009.pdf]

Table S3. Polyunsaturated fatty acid composition of offspring aorta total lipids on day 77

	Fatty acid concentration (% total fatty acids)							
	Maternal dietary group							
	SAO		HSO		Butter		MO	
	7%	21%	7%	21%	7%	21%	7%	21%
	Male							
n	6	6	6	6	6	6	6	6
18:2n-6	13.6±2.9	15.1±2.5	11.7±2.4	11.4±2.0	12.2±0.7	10.3±2.0	9.4±0.9	12.5±3.1
20:3n-6	0.5±0.2	0.3±0.1	0.4±0.4	0.2±0.8	0.3±0.2	0.1±0.1	0.5±0.3	0.2±0.1
18:3n-3	0.1±0.5[a]	1.0±0.9 [b]	0.3±0.2[a]	1.1±0.3 [b]	0.2±0.2[a]	1.0±0.3 [b]	0.2±0.1 [b]	1.2±0.2 [b]
20:5n-3	0.2±0.1	0.2±0.1	0.2±0.1	0.1±0.8	0.2±0.1	0.2±0.2	0.2±0.1	0.2±0.5
22:5n-3	0.5±0.1	0.4±0.2	0.5±0.2	0.6±0.9	0.3±0.2	0.2±0.1	0.6±0.2	0.4±0.1
	Female							
n	6	6	6	6	6	6	6	6
18:2n-6	11.1±1.5	14.7±2.8	9.6±1.4	12.5±3.6	12.2±0.7	12.5±3.6	11.0±2.5	12.9±3.3
20:3n-6	0.7±0.3	0.4±0.1	0.6±0.2	0.3±0.1	0.6±0.2	0.3±0.1	0.7±0.3	0.5±0.1
18:3n-3	0.2±0.1[a]	1.2±0.5 [b]	0.2±0.1[a]	1.2±0.3 [b]	0.2±0.2[a]	1.9±0.3 [b]	0.1±0.1[a]	1.0±0.2[b]
20:5n-3	0.1±0.1	0.2±0.1	0.1±0.0	0.1±0.1	0.2±0.1	0.2±0.1	0.3±0.2	0.2±0.0
22:5n-3	0.4±0.2	0.3±0.1	0.5±0.2	0.6±1.0	0.3±0.2	0.3±0.1	0.9±0.5	1.0±0.8

Values are mean ± SD proportions of fatty acid in individual maternal plasma lipid classes (n = 6 offspring per group). Different superscripts indicate values which were significantly different ($P < 0.05$) by a general linear model with Tukey's *post hoc* analysis. The proportions of 20:4n-6 and 22:6n-3 are reported in Figure 3.
