# Supplementary material for: Vascular Dysfunction Induced in Offspring by Maternal Dietary Fat Involves Altered Arterial Polyunsaturated Fatty Acid Biosynthesis
Source: PLoS One. 2012 Apr 3;7(4):e34492. doi: 10.1371/journal.pone.0034492 (PMC3317992; doi:10.1371/journal.pone.0034492)
Supplement: Figure S3 — Sequence of the 5′ regulatory region of rat Fads2 . CpG dinucleotides are indicated by bold text. Numbers above the text indicate the locations relative to the transcription start site (bases) of individual CpG dinucleotides which were measured by pyrosequencing. Underlined text indicates the location of a putative estrogen receptor response element. Arrow marks the transcription start site. (PDF) [file pone.0034492.s003.pdf]

5’
TTTTATGTGGTGATTATTT

- 722
- 780 ATTTTTTTTATTTTTTTGTGTATTTTTTGATGTGGTTAGATTGAGTGTGTTTTTGTTT**CG**

- 711 - 678
- 720 TTTTTGGTA**CG**AGAGATATTAAAATGTAGAATATTATAGTAA**CG**TTAGGGAAAGTTTGTA

- 623 - 605
- 660 AATTTATGAGAATAAAATGAAGTGTAATTTATGTTTT**CG**GTATTAGTTTAATTTT**CG**AAA

- 585
- 600 TTTTGTGTTAGGTTT**CG**TAGGAATTTTTTTATATGATTTTATGTTATTTTTTTTGTTGTT

- 540 GTTTTATGATATTTAAATATATTTTTTAGGTTGTAGTTATTTTTTAGGTGTTAGGGATTG

- 480 AGTTGGG**CG**TTTTATATATGTTGGGTAAATATTTTTATTATTGAGTTAAATTTTTTTTT

- 394
- 420 GAGTTTTATTTTTTTATTGATAATTT**CG**ATTTTGTGGAAAAGGATATTTTTGGAGTGGTG

- 360 GGTGGTATAGTTTGTATATTGAAGGATATTTAGTTTAGGAGTAGGTTAGGGGTGTTTTTTT

- 300 TGAGTTTTGTGTTTATTTGTTTTTTTTGGAGATTTATTTGTTTATAAATTTTTGATTAGT

- 240 TTAGTAGGTTTGGTGTTAGTTTAGAGGTTTAGGATGGTTATGGTTTGTTATTAGAGTTTT

- 180 GTATTGGGGATATGTTTTGTGGTGGAGTTTGGAGTAAGTATTTGTTATTGAGGAGTGGGA

- 84 - 76 - 65
- 120 AGTTTTATTTGGTTTTTGAGTAGATATTGGATTTG**CG**GTAAAA**CG**TTTTTTTTT**CG**AGAG

- 31
- 60 TGGGAGGAGTTAGTATAGGTTTGGGGTT**CG**GTTTTAGTAGTTTGTTTTTTTGTTTTTTAG

GATGTTTTT
3’

Figure S3. Sequence of the 5’ regulatory region of rat *Fads2*. CpG dinucleotides are indicated by bold text. Numbers above the text indicate the locations relative to the transcription start site (bases) of individual CpG dinucleotides which were measured by pyrosequencing. Underlined text indicates the location of a putative estrogen receptor response element. Arrow marks the transcription start site.
