# Supplementary material for: Vascular Dysfunction Induced in Offspring by Maternal Dietary Fat Involves Altered Arterial Polyunsaturated Fatty Acid Biosynthesis
Source: PLoS One. 2012 Apr 3;7(4):e34492. doi: 10.1371/journal.pone.0034492 (PMC3317992; doi:10.1371/journal.pone.0034492)
Supplement: Figure S5 — Sequence of the 5′ regulatory region of rat Fads1. CpG dinucleotides are indicated by bold text. Numbers above the text indicate the locations relative to the transcription start site (bases) of individual CpG dinucleotides which were measured by pyrosequencing. Arrow marks the transcription start site. (PDF) [file pone.0034492.s005.pdf]

5’

- 120 TCCCACACCACT**C**GGCTCAGCCAATGGGAGGG**CGCG**C**CGCG**GGGCTTGGAGGTC**CGCG**CC

- 39 - 33 - 23 - 15 - 9

- 41 - 37 - 30 - 17 - 11 - 1

- 60 AA**CG**GTGCTGAAAACCCTG**CGCGCG**AA**CG**G**CG**GTCTC**CG**GGAG**CGCG**CT**CGCG**GCAGCC**C**

GACTCT

3’

Figure S5. Sequence of the 5’ regulatory region of rat *Fads1*. CpG dinucleotides are indicated by bold text. Numbers above the text indicate the locations relative to the transcription start site (bases) of individual CpG dinucleotides which were measured by pyrosequencing. Arrow marks the transcription start site.
